# Methods for assessing small-scale variation in the abundance of a generalist mesopredator

**DOI:** 10.1371/journal.pone.0207545

**Published:** 2018-11-21

**Authors:** Jim-Lino Kämmerle, Luca Corlatti, Laura Harms, Ilse Storch

**Affiliations:** 1 Chair of Wildlife Ecology and Wildlife Management, University of Freiburg, Freiburg, Germany; 2 Forest Research Institute of Baden-Württemberg FVA, Freiburg, Germany; University of Minnesota, UNITED STATES

## Abstract

Estimating animal abundance is essential for research, management and conservation purposes. Although reliable methods exist to estimate absolute density for populations with individually marked animals, robust relative abundance indices (RAIs) may allow to track changes in population size when individual identification is not possible. Their performance, however, needs be thoroughly evaluated. We investigated the relative performance of several common faeces-based and camera-based RAIs for estimating small-scale variation in red fox abundance, a mesopredator of high relevance for management, in two different study areas. We compared precision, cost and performance of the methods in capturing relationships with covariates of local abundance. Random transect-based RAIs had a low mean, a comparatively high coefficient of variation and a high proportion of zeros, prohibiting or impeding analysis in relation to environmental predictors. Rectangular scat plots and transects along linear landscape features had an intermediate amount of zeros while retaining a high precision, but were less sensitive to local variation in abundance related to environmental predictors and required a large field effort. Camera trap-based RAIs yielded low to intermediate precision, but were more sensitive to small-scale variation in relative abundance than faeces-based methods. Camera traps were the most expensive methods for an initial monitoring session, but required the lowest field effort, were cheapest in the long run and were the least susceptible to observer bias and detection error under a robust sampling protocol. Generally, faeces count-based RAIs appear more suitable for studies that aim to compare local abundance between several study sites of equal landscape composition under constant detection probability. Camera traps provide more flexible data for studies that require accounting for influences of landscape composition on local abundance and are more cost-effective for long-term or continuous monitoring and more suitable to achieve high replication. Accordingly, the choice of the most suitable method and plot design is context-dependent.

## Introduction

Acquiring knowledge on size, distribution and dynamics of animal populations is pivotal for research, management and conservation issues [1,2]. To assess the effectiveness of conservation actions it is essential to implement efficient monitoring of population size [3]. Monitoring should not only aim to estimate variation in abundance of the target species across large areas (i.e. landscape scale; [4]), but also differences in abundance between restricted areas (i.e. small scale), because local management actions at the district or community level require information collected at small spatial scales, typically ranging in extent from hectares to few km^2^. Reliable estimates of absolute abundance, however, may be difficult to obtain even for common species.

Capture-recapture methods may provide robust estimates of local density for species that allow for individual identification [1,5,6], yet owing to the high costs they are potentially unsuitable for long term studies. In recent years, a number of methods to estimate absolute density without the need for individual identification have emerged [7–9], yet they also rely heavily on sets of assumptions or have not been sufficiently validated. In these circumstances, the use of robust population indices may present an alternative to track changes in population size [10]. Indeed, for species that are not individually identifiable, researchers often resort to indices that quantify differences in relative abundance based on a variety of field signs (i.e. relative abundance index, RAI [2]). Comparison of RAIs is, however, limited by differences in detection probability across indices, space or time [2,11]. Ideally, the relationship of the index with real abundance in different contexts should be known or at least predictable, so that differences in detection probability can be accounted for in research design and suitable RAIs can be selected. Evaluating the relative performance and context-specific applicability of RAIs before commencing a survey is thus paramount [11], though this is seldom done in practice, nor always feasible.

All RAI methods share the common assumption that the index measured is directly related to the property of interest (i.e. absolute abundance), but the exact shape of this relationship is often unknown [2,11]. Consequently, different RAIs should exhibit similar patterns in relation to environmental covariates (less the method-specific noise) when applied to the same population of animals, provided that the covariates themselves are related to true abundance and that the indices respond to variation in the covariate within the gradient studied. While this assumption is reasonable in the presence of a linear relationship between RAI and true abundance, it might be problematic in the case of non-linear relationships as variation in abundance related to the covariate might not be reflected by the index. In addition, an ideal RAI method should have high precision (i.e. narrow dispersion) to maximize chances of detecting differences if present (e.g. between management units), whilst being sensitive to variation in the underlying process across space or time (i.e. animal abundance). Furthermore, variability should also be large enough to allow for adequate representation of relationships with environmental covariates and the data should have a low proportion of ‘false’ zeros (unless abundance is truly zero) in order to facilitate analysis. Finally, labour-intensive methods should be simple enough to ensure efficient use of volunteer field assistants [12] to allow for adequate replication.

Predators are often of high priority in wildlife management, either owing to their conservation status (e.g. large felids: [13–15]), or because of their impact on threatened prey [16,17] or on valued game species [18]. For the red fox (*Vulpes vulpes*), a widespread mesopredator of global relevance for conservation and wildlife management [19], individual animals are difficult to identify non-invasively [20] and, consequently, RAIs are often employed. For example, sampling along trails or other linear features is a common method to estimate fox abundance [21–23], although camera-based RAIs (e.g.[24]) or spotlight counts (e.g. [12,25]) have also been employed. Some studies have evaluated the performance of several RAIs to estimate landscape-scale variation in fox abundance [12,24,26], but their performance is unknown when applied to small-scale studies, i.e. where the variance in fox abundance is low because the overall environmental gradient covered by the study is small.

In this paper, we aim to compare the relative performance of different RAI methods–faeces count methods and wildlife camera-based RAIs, both of which are thought to be representative of real abundance [23,24]–to measure small-scale variation in the abundance of red fox. We use data from extensive field-experiments in two different environmental contexts: montane forests and agriculturally dominated landscapes. We assessed the performance of the respective methods by comparing data quality (dispersion, percentage of zeros), the method’s ability to detect and reflect relationships with environmental covariates of abundance as well as cost and effort. Our results shall provide useful indications on how to collect robust data for red fox ecology research and population monitoring, thus helping to evaluate the effectiveness of management interventions for this common generalist mesopredator.

## Methods

### Study area

**Fig 1:**
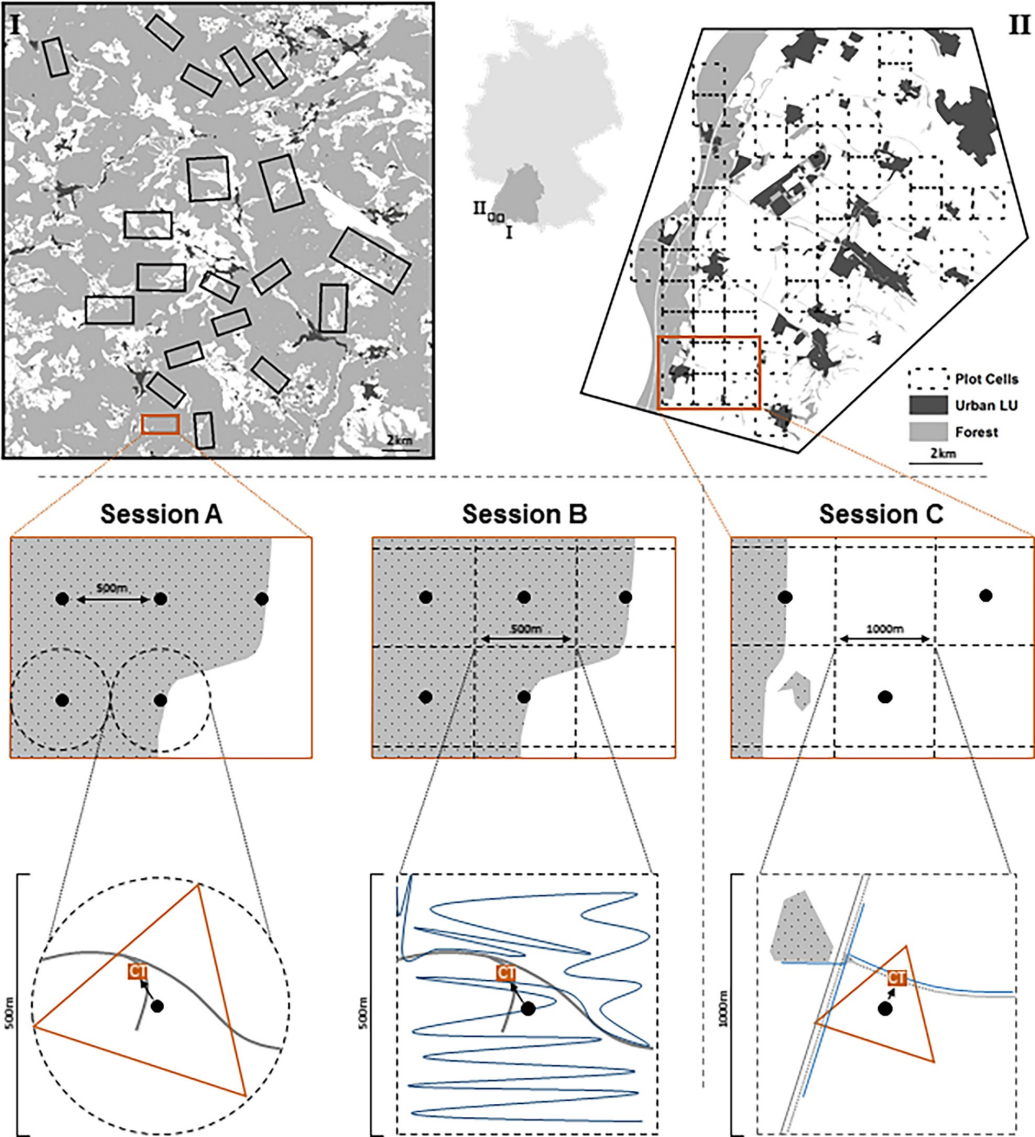
Schematic representation of the experimental design used to compare different methods to estimate variation in red fox abundance in the Black Forest mountain range (I; session A–March-May 2017 & B–March-May 2018) and in the agricultural lowlands of the Upper Rhine Valley (II; session C–November 2017 and February 2018) in south-western Germany. In each session, we compared RAIs obtained from grid-based remote camera traps (rectangular box labelled ‘CT’) with triangular random faecal transect counts (session A and C, orange), scat-plot based faecal counts standardized by search duration (session B, blue) and faecal transects of fixed length along linear landscape features (session C, blue). Grey lines represent forest tracks in session A & B and linear landscape features in session C (e.g. hedges).

Two study areas in South-Western Germany were chosen to represent different landscape types, one comprising montane forests, the other agriculturally dominated lowlands. The montane forest study sites were located in the southern Black Forest mountain range (Fig 1) at altitudes of approximately 900 to 1300 m. The area is characterized by mixed montane forests of spruce (*Picea abies*), silver fir (*Abies alba*) and beech (*Fagus sylvatica*) trees [27], interspersed by settlements and small towns surrounded by extensive mountain pastures, thus creating a forest dominated land use matrix (Fig 1). The agricultural study sites were located in the lowlands of the Upper Rhine valley between the cities of Basel and Freiburg along the river Rhine in the West (Fig 1) at a maximum elevation of 220 m. Land use and cover is mainly agricultural, with large continuous stretches of farmland (mainly cropland, some pastures, meadows and orchards) accounting for about two thirds of the landscape. The remainder consists of urban land cover types and a continuous stretch of riparian forest along the river Rhine (Fig 1). There are no reliable estimates of absolute red fox density for the study areas, but home-range sizes obtained through VHF telemetry in a valley of the montane forest study site (100% MCP, $\bar{x}$ = 197 ha; range 62–378 ha [28]) suggest intermediate fox density [29]. Effective monitoring of red fox populations has immediate management relevance in both study areas, as red foxes are considered important predators of locally threatened prey species: the capercaillie (*Tetrao urogallus*) in the Black Forest [30] and the European hare (*Lepus europaeus*) as well as a number of ground nesting birds in the Upper Rhine valley [31].

### Study design

To evaluate the suitability of different methods for estimating small-scale variation in the relative abundance of red fox, we conducted three sets of paired experiments with a total of six different methods (Fig 1) between March 2017 and May of 2018. For each set of experiments we compared the performance of different types of methods: faeces-count methods (either using random transects, rectangular scat plots or transects along linear landscape features) and a wildlife camera-based method (Fig 1). Fieldwork was conducted in winter and early spring (i.e. in November or between February and May) in order to obtain an estimate of red fox winter abundance before new offspring contribute to the population size. A comprehensive summary of the methods used is included in the supplements (Table A in S1 Appendix). This study did not involve capture, handling or any other form of invasive interaction with animals. It was carried out in strict accordance with national legislation. Permission of landowners was obtained prior to commencement of the study.

#### Montane forest

For our Black Forest study area, we surveyed populations of red foxes in the spring of 2017 and 2018 using three relative abundance indices (RAIs) along a network of spatially coinciding plot locations. In the spring of 2017 (henceforth: ‘Session A’) we applied random faecal transects paired with a plot-point based network of camera traps. In the spring of 2018 (‘Session B’) we combined plot based wildlife cameras with faecal counts within rectangular plot areas standardized by search duration.

For Session A, we chose a set of rectangular study sites within the study area to be representative of the landscape composition in the area and excluding large built up areas. Study sites directly covered approximately 6.500 hectares (ca. 32% of the study area). In a second step we assigned circular plots within the study sites to orient placement of random transects and camera traps using a systematic grid of 500 m spacing. We distributed survey effort proportionally across the study sites stratified by expected fox abundance (fox abundance based on camera model in [24], predictions as in [30]).

At each plot, we conducted faeces count surveys along random triangular transects centred on each plot location (i.e. transects never overlapped between plots, Fig 1). Faecal sampling along trails and other linear features has been a standard field method to estimate fox abundance [21–23], but foxes only deposit a minority of scats along linear features [22]. Transects along such features have thus been criticised due to the inherent bias with regards to selective habitat coverage and lower precision than random transect sampling [26]. We therefore adapted the linear random transect method [32] for application on a smaller spatial scale. Triangular transects were 1.3km long and randomly oriented, equalling the maximum triangle size in a 250m radius circular plot (i.e. half the distance between plot centres). In general, faeces abundance of foxes is thought to be representative of real abundance [23,24]. Transect counts for spring abundance were conducted in spring (29.03.2017 and 10.05.2017) after snow melt and before ground vegetation (mainly *Vaccinium myrtillus*) interfered with search efficiency. Transects were walked at a speed of approximately 1km/h and all scats spotted by the observers were collected. We identified fox faeces according to their size, shape, odour and content [33]. Species with potentially similar scat in the study system are pine (*Martes martes*) and beech marten (*Martes foina*), which both typically produce scats that are much smaller, and dogs. For this method as well as other scat-based methods in this study area, we applied multi-observer validation for scat identification (i.e. each scat was validated to be fox scat by at least one expert in addition to field personnel). We randomly assigned observers to scat plots within the study areas (session A&B: 6 observers; session C: 3 observers). In addition, we scored scats along a gradient of scat quality and identification certainty using three classes (A-C, A being highest).

We additionally surveyed red foxes using non-baited remote, heat and motion triggered cameras (Bushnell Trophy Cam Aggressor Low Glow) placed at plot locations (Fig 1: symbol ‘c’). Camera surveys of fox abundance have been shown to deliver comparable estimates to scat transects and thus to be related to real abundance, albeit at higher variance [24]. To standardize detection probability across all sample plots and to maximize red fox detection rates we placed one camera per plot in the same fashion on trees along machine roads and logging tracks [24]. We selected the closest suitable track outwards from the plot centre. Camera orientation was slightly angled to ensure good coverage of the track and detect fast-moving individuals with higher certainty. All efforts were made to select similar tracks within each plot. We also recorded trail width and trail class (4-level factor: 1 = game trail or almost completely overgrown logging track; 2 = non-maintained logging track with young vegetation; 3 = maintained machine track, 4 = unpaved forest road) to correct for differences between the probability of trail use by foxes among trail types. In addition, we also quantified the percentage of ground in a 20m plot around the camera covered by structures that would hinder fox movement and thus channel foxes onto the tracks (i.e. dense vegetation <1m, coarse woody debris, large rocks; abbr.: ‘%Resistance’). Cameras were placed in the field for at least 21 operational camera days before collection. Cameras were deployed in two consecutive sessions of N = 70 each between 17.03.2017 and 12.05.2017. We set cameras to take sequences of three pictures with a 1-second delay between series. Final sample sizes were N = 132 camera sites and N = 114 random transects for session A.

For session B we also used a grid of 500m spacing for plot allocation. However, instead of sampling faeces along random transects we searched for fox faeces within quadrat grids (i.e. 500x500m cells; 25ha each; henceforth ‘scat plots’). Scat plots were surveyed hap-hazardly aiming for representative plot coverage, but search effort was standardized by searching for three man-hours on each plot. Trail-based sampling has low precision, whilst sampling within small grid cells (0.25ha) was found to return a very small mean (i.e. large proportion of zeros [26]). Accordingly, we designed larger scat plots to ensure a larger mean and higher precision by representatively sampling the whole plot (i.e. including tracks as well as all available habitat types). Due to the higher field effort involved compared to random transect sampling, we concentrated our field effort on a subset of four study sites of 10km^2^ size (i.e. 4.000 ha), each including approximately 20 plots (overall sample size N = 82) and covering the same landscape gradient as in Session A. We surveyed all scat plots between 03.04.2018 and 18.04.2018 before ground vegetation could interfere with search efficiency and while some plots still retained partial old snow cover to maximize the detection of fresh scats. Scat identification was validated and scored as described above.

As for session A, camera traps were deployed in all study sites (final N = 151 plots), including the N = 82 scat plots. Cameras were deployed following the same protocol as in session A between 15.03.2018 and 16.05.2018.

#### Agricultural lowlands

For our agricultural study area in the Rhine valley (‘Session C’) we used a systematic grid array with 1x1km cell size for plot allocation to account for a more continuous land cover composition (i.e. less turnover) as compared to the forest study area. We selected a total of 40 plots representative of the landscape composition in the study area (total area covered by plots: 4000ha). Within each plot we compared three different methods in two rounds of field work in the winter of 2017/2018: one in November 2017 and another in February 2018, when ground vegetation and standing agricultural crops on farmland were minimal, thus ensuring high visibility.

For the first method we conducted scat surveys along random triangular transects in each plot with the same method and survey protocol as for session A. When transects partially overlapped with urban areas, they were moved away from the settlement until no overlap occurred to improve accessibility. Random transects were conducted between 20.11.2017 and 24.11.2017. In this study area, we used fewer, more experienced observers and thus only applied expert validation for scats considered uncertain.

As a second method, we conducted a faeces count along linear transects oriented alongside linear landscape features, a sampling strategy that has been used previously to monitor fox abundance (cf. [22]). Transects of 1.3km in length (equal to the random transect length) were allocated beforehand within each plot (N = 40) using GIS software along linear landscape structures such as groves, hedges, ditches and forest edges, identified using geodata and aerial imagery. Unpaved and paved roads were avoided due to the large differences in availability within each plot and the high presence of dog walkers (and associated potential of scat misidentification). Apart from transect allocation, the field protocol was analogous to random transect sampling. Transects were conducted between 06.02.2018 and 08.02.2018.

Finally, we deployed one camera trap at each plot in both rounds as a third method and left it operational for at least 21 days between 14.11.2017 and 13.12.2017 and between 31.01.2018 and 23.02.2018, respectively. Cameras were placed outwards from the plot centre along the closest suitable linear landscape feature (i.e. groves, hedges, ditches and forest edges): this design allowed to standardize detection probability and enhance encounter rate, as foxes were hypothesized to use such structures for movement within otherwise very open landscapes. A different site was chosen in the second round to maximize the coverage of environmental variability and reduce site effects. Cameras were set up at approximately 20cm above the ground on trees or using wooden poles if no trees were present. At each location we recorded the type of linear structure (four types), the type of ground cover at the camera plot (five types) and proximity to roads (paved and unpaved) to control for potential differences in detection probability among sites. Sample sizes for session C were N = 77 (38 + 39) camera sites, N = 40 random transects and N = 40 transects along linear features.

### Data preparation

For transects and scat plots during all sessions, we summed the number of faeces found per plot ID to obtain the faeces count-based RAIs. Scats of all three classes (i.e. A-C) were considered in the RAIs.

With regards to camera trap data, we downloaded images from each camera in each study session and sorted images at the species level. We then extracted the image metadata using the package camtrapR [34] in R [35] and grouped image sequences into events using a break value of 5 minutes between images as a conservative value based on visual assessment of the images. All fox events were exported and assigned to their respective plot ID before being summed as the number of fox events per trap station. For each camera, we finally calculated the number of active trap nights (i.e. nights that the camera was operational in the field) using the recorded images to determine periods during which cameras were not operational. This procedure was applied to all camera traps during all sessions.

#### Environmental predictors

We assigned environmental predictors to all our RAIs at the spatial scale of the plot.

For session A, a circle of 250m radius around camera locations and transect centres was chosen (i.e. area of 19.6 ha), since this is the smallest circle containing the transect triangles as well as the half-distance between plot centres. We did not choose a larger radius to avoid overlap between plots as we were aiming to depict local variation in abundance. For session B, we used the size of the quadrat scat plot (i.e. 500x500m, area of 25 ha) to assign environmental data to the scat plots, but for the camera method we followed the procedure as in session A. For both sessions we extracted the elevation above sea level, the mean distance to human settlements of the plot (including single farms) and quantified landscape heterogeneity using the Shannon Index [32,36] with the proportions of the four land cover types in the study area (i.e. forest, pasture, arable, settlement) at the scale of a mean fox home-range in the area (i.e. 200ha, r ≈ 800m; [28])

Similarly, for session C a 250m buffer was defined around each camera location. Faecal counts were, however, processed differently to better compare both transect methods within plots of session C. We extracted environmental covariates in a 75m buffer around the transect lines for both transect types (i.e. aiming for a buffer size of 20ha as for the camera method). Due to the different landscape composition and terrain of the study area in session C (i.e. no elevation difference, different landscape matrix), we extracted the proportion of the plot covered by forest and settlements, respectively, and calculated the Shannon Index of landscape heterogeneity as described above.

### Data analysis

We assessed the performance of the different methods in all three seasons with respect to their ability to detect and reflect relationships with environmental covariates of abundance, the quality of the resulting data (measured as: dispersion, percentage of zeros), as well as the cost and effort of the method.

#### Relationships with environmental covariates

We analysed our RAIs from all sessions in dependence of an environmental covariate known to be related to fox abundance to evaluate the ability of the method to reflect and test for differences in habitat and landscape related patterns and assess whether the resulting patterns corresponded well amongst the methods. We used the Shannon Index of land cover diversity, as this predictor is known to be closely related to variation in both camera- and scat-based RAIs for foxes in our study area [24,32]. We fitted generalized linear models (GLM) with negative binomial distribution of errors and the abundance index as a response for each RAI method. The Shannon Index of land cover diversity was included into each model for comparison across methods. We also included a set of additional environmental predictors at the plot scale into each global model to control for differences in relative abundance between plots. For sessions A and B we included the elevation of the plot and the mean distance of the plot to human settlements into each global model. For session C we included the proportion of plot area covered by settlements and forests into all global models. A number of further control variables were included to account for differences in detection probability between plots. For the camera-based RAIs: the type of track at the trap site (large track vs. small trail) and the ‘%Resistance’ around the camera site (r = 20m) in session A & B and the type of linear landscape feature used for camera placement (four types), the type of ground cover at the camera plot (five types) and the round of fieldwork (November vs. February) for cameras in session C. For all camera methods we also included the number of active trapnights as a model offset. For the scat-based RAIs we included observer ID into the models to account for differences in detection probability between observers. We standardized all continuous predictors by subtracting the mean and dividing by the standard deviation to allow for comparison of effect sizes. GLMs were fitted using the MASS library [37]. We then performed AIC-based stepwise model selection on each global model selecting the model with the lowest AIC value while holding the predictor Shannon diversity fixed in all models. We calculated Pseudo-R^2^ for each method as $R^{2}=1-\frac{Residual\;Deviance}{Null\;Deviance}$ as a coarse indication of variance explained by our covariates.

#### Data quality

For each RAI, we measured data quality using variability and precision (i.e. dispersion of the data) and the proportion of zeros in the dataset. In order to assess the dispersion of the data we quantified the mean and variance of the negative binomial distribution estimated by the models. The variance of the negative binomial distribution can be defined as *var*(*Y*) = *μ* + *α* * *μ*^2^ [38] and the dispersion parameter α as $\alpha =\frac{1}{\theta }$, with θ being estimated by the model: Accordingly, the data exhibit larger spread around the mean with increasing α and increasing mean. To compare precision between methods we report the α parameter and calculated the coefficient of variation (CV) as $CV=\frac{\surd var(Y)}{\mu }$, since data were on different scales and the CV provides a standardized (i.e. dimensionless) measure of dispersion. We plotted the empirical probability density function in comparison to the negative binomial function predicted by the model. We also calculated the proportion of zeros (i.e. no fox signs detected at a plot) for each RAI method.

#### Cost and effort quantification

We quantified field effort and costs based on the mean costs of conducting a single plot and then standardized cost estimates to an effort of 100 plot sites for each method by multiplying the costs. We assessed the effort as the mean number of staff working hours per plot and the cost as the sum of material costs and wages for each plot. We used a wage estimate of 240€ per day (8h) for a qualified worker in our study region [24]. We used 250€ as a price estimate for a mid-range motion triggered infrared wildlife camera including accessories (e.g. SD card, locks). We included working hours for post processing (i.e. downloading and identifying fox images) into each estimate, which averaged 0.25h / camera trap for processing and sorting images (this estimate is likely quite low, owing to the comparatively small number of pictures/trap in our data; Table C in S1 Appendix) and 0.1h / transect or scat plot for multi-observer validation of faeces. Transportation costs to and from the plot sites as well as working hours needed to delineate plot locations were assumed to be equal across methods and are thus not included in our estimates. We provide cost estimates for an initial sampling session (i.e. including full initial material costs) and a subsequent session with existing technical equipment and only running equipment costs (i.e. mainly labour costs).

## Results

**Fig 2:**
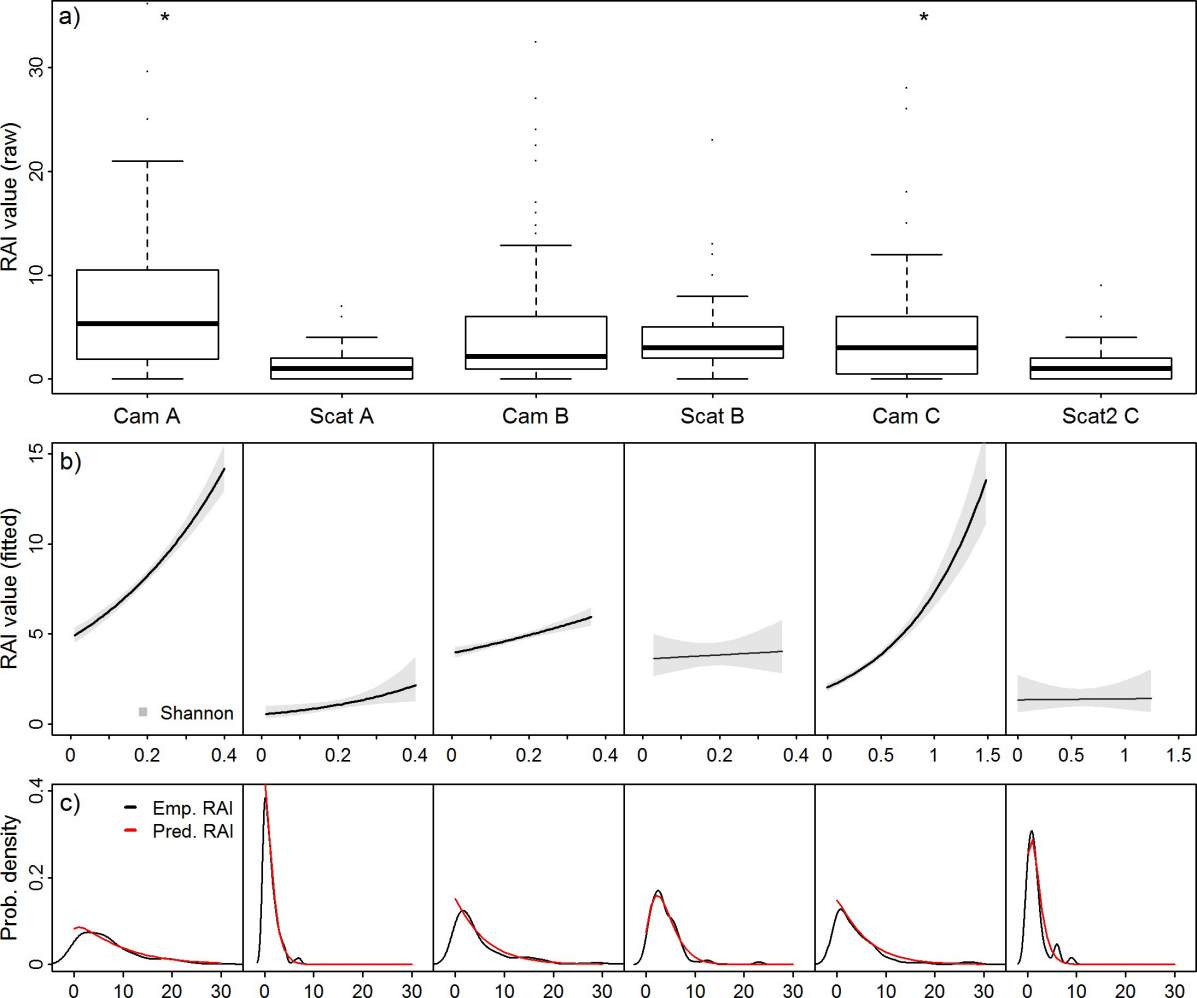
Overview of the results obtained by the methods in each session. Each column corresponds to one method with letters representing the sessions A-C, while rows contain different evaluation metrics for the RAI methods. a) Boxplot of the distribution of raw RAI values (camera data standardized to 21 trapnights); b) Conditional effect plots of the relationship of the predictor Shannon Index of land cover diversity (x-axis)with the RAI as predicted by the final models, with all other predictors set to the mean. A bold line indicates a significant effect in the model. Note the different range of Shannon values in landscapes of session C; c) Empirical and predicted probability density functions for the RAI values (x-axis) obtained from each method. Final models for each method were used for the predicted PDF assuming a negative binomial distribution of the data. *Y-axis scaling in a) omits 4 (Cam A; 3.0%) and 2 (Cam C; 2.6%) RAI values > 36.

Table 1 provides an overview of dispersion, data quality and cost per plot. Overall, camera data had larger means and thus larger variance than the faecal count methods during all sessions in both study areas (Table 1; Fig 2). This is reflected by a larger α parameter for camera data than faeces-based methods, but this pattern is not evident for the coefficient of variation. Random transects had the lowest mean and the highest percentage of zeros in the data, regardless of the study area (43.9 and 50.0%; Table 1). Scat plots and transects along linear features had the smallest α values (Table 1). CVs were highest for random transects in session A and camera traps in session B and C. Cameras in session A and transects along linear features in session C had an intermediate CV. CV was lowest for the scat plot method.

**Table 1:** Descriptive statistics on dispersion and effort for each method in all three sessions. Variance estimates are provided for the raw data (naïve) and as obtained from the final models assuming a negative binomial distribution (negbin). Cost per plot is estimated as the sum of costs for material and personnel (excluding travel; see method section) and provided for both a first session and any consecutive session. A more comprehensive version of this table can be found in Table A in S1 Appendix. Note that the methods have different units: Camera traps (CT): number of fox events over 21 days; Random and linear transects: number of scats per transect; Square scat plots: number of scats per plot.

	Session A		Session B		Session C		
	**CT**	**Random Transect**	**CT**	**Square Scat Plot**	**CT**	**Random Transect**	**Linear features**
Mean	7.94	1.12	4.38	3.88	4.36	0.68	1.68
Median	5.46	1.00	2.17	3.00	2.86	0.50	1.00
Var (naïve)	81.4	2.10	31.1	11.1	45.7	0.64	4.02
Var (negbin)	67.3	1.89	32.5	8.21	30.7	-	2.46
θ (negbin)	1.20	1.65	1.04	3.47	1.08	-	3.56
α (negbin)	0.83	0.61	0.96	0.29	0.93	-	0.28
*CV*	1.03	1.23	1.30	0.74	1.27	-	0.93
Min	0	0	0	0	0	0	0
Max	59	7	34	23	50	3	9
% Zeros	6.8	43.9	17.8	4.9	24.7	50.0	27.5
Hours / plot	0.6	1.6	0.6	3.1	0.6	1.4	1.4
Cost / plot (1^st^)	270.4€	49.5€	270.4€	94.5€	270.4€	43.5€	43.5€
Cost / plot (2^nd^)	20.4€	49.5€	20.4€	94.5€	20.4€	43.5€	43.5€

Costs per plot were lowest for random transects for an initial sampling session (Table 1). Costs for camera trapping were, however, much lower than for all other methods in subsequent sessions, once equipment costs were removed (Table 1). Average costs for 100 plots were thus €27040 for camera trapping, €9450 for scat plots and €4950 (session A) or €4350 (session C) for transect in the initial session. Costs were reduced to €2040 for camera trapping in each subsequent session, while they remained the same for all other methods. In session C, 3.75% of the traps were stolen, representing an additional cost of €9.38 for each plot or €938 for each consecutive session of 100 plots. No camera was stolen in session A and B.

### Model results

The negative binomial probability density distributions estimated by the models fit the empirical data distributions well (Fig 2), with the exception of random transects in session C, where data quality did not allow model convergence (i.e. 50% zeros in the data). Accordingly, we only provide model results for the remaining six methods in three sessions. An overview of the final models is shown in Table 2. The Shannon Index of land cover diversity had a positive relationship with the RAI for each method and both study areas, but the slope was not significant for the scat plot method in session B and transects along linear features in session C (Fig 2). The effect size of the Shannon Index was larger for camera based RAIs than for transect methods (regardless of the study area) and largest for the camera model in session C, followed by session A (Fig 2). Elevation had a significant negative relationship with RAIs in session A and B, except for the random transect method in session A for which the final model only included the Shannon Index. There was a significant but small negative effect of distance to settlements in the final model of camera RAIs in session B. The final model for camera RAIs in session C included a significant negative relationship with the proportion of urbanized area in the plot and a significant positive relationship with the proportion of forest in the plot. The effect of forest cover was retained in the final transect model for session C with a negative slope, but the slope estimate was not significant.

## Discussion

In this study, we compared the relative performance of several commonly used RAI methods in two study landscapes, montane forests and agricultural lowlands, with respect to data quality, cost and the ability to depict small-scale variation in fox abundance in relation to selected environmental characteristics. A decision tree for choosing the appropriate method in specific contexts based on our findings is included in the supplements (Figure A in S1 Appendix).

While mean and variance were lower for scat-based than for camera-based methods, surprisingly there were no clear differences in precision between the methods as indicated by the coefficient of variation, in contrast to previous work [24]. When random transects were used in study sites similar to ours, they yielded high precision and offered the possibility to depict significant differences in RAIs between study areas [26]. Furthermore, in our study low means were associated with a high proportion of zeros for random transects in both sessions. By contrast, camera data and linear transects in session C had comparable (moderate) percentages of zeros, while scat plots and camera data in session A and B were characterized by low to intermediate amounts of zeros. However, assumptions of equal detection probability across different sites when comparing RAIs are problematic [11], thus we refrained from testing for differences in absolute RAIs between study areas and limit our evaluation to comparison of different performance metrics. Nonetheless, mean RAIs were comparable for random transects in the Black Forest and transects along linear features in the Rhine valley, but the linear feature method had a more balanced empirical distribution (Fig 2C) and fewer zero values. The random transect method in the Black Forest successfully captured variation in abundance related to landscape composition, albeit with a very small effect size, whilst transects along linear features did not, even though the range of the landscape predictor (i.e. Shannon Index) was much larger in session C (Fig 2). Conversely, random transects in session C were characterized by a very low mean and an extreme proportion of zero values in the dataset (i.e. 50%), to a degree that was prohibitive for analysis. This value would have increased, if only scats of high identification certainty had been included in the analysis. Since foxes occur anywhere in both study areas, zeros in the dataset most likely represent ‘false’ zeros (i.e. failure of the method to detect the animal). This is supported by successful fox detection by the camera traps in those plots where no scats were found (e.g. in session A 42 of 46 zeros identified as false zeros by cameras in the same plot). While zeros are an intrinsic realisation of the assumed probability distribution and not per se adversely affecting analyses, excessive amounts of ‘false’ zeros strongly reduce the information content of the data, impose unnecessary burdens of factoring in (potentially immeasurable) control variables of detection probability and thus challenge the appropriateness of a method given the high field effort involved. The amount of zero in the data will, however, be related to the size of the plot searched for faeces. This is reflected in the results of the scat plot method, which had larger means and higher precision when compared to the other faeces count-based methods, resulting in a mean similar to camera data, but at higher precision (Table 1, Fig 2C) and the lowest proportion of zeros in the dataset. This method was, however, not sensitive to the environmental predictors used in this study (except elevation; Table 2). While this may be related to the spatial scale of the plot, scat plots were only 20 per cent larger than camera plots and random transect plots (i.e. 20ha vs. 25ha), which were both able to capture a significant relationship of local abundance with landscape heterogeneity in the Black Forest (session A&B; Table 2). While both cameras and faeces-based RAIs responded well to environmental covariates when studying landscape scale differences in abundance [24], in our study only camera traps were able to always capture variation in local abundance related to landscape heterogeneity in both study areas (Table 2, Fig 2B). Overall, study sites within the Black Forest study area covered a relatively small gradient of landscape heterogeneity (i.e. compare predictor ranges in Fig 2B). Therefore significant relationships reported by the models are likely reflecting local, small-scale variation in abundance. Faeces-based methods generally appear less capable of capturing this local variation (i.e. small effect for random transects, no effect for scat plots), potentially indicating that scat deposition is either random and thus not indicative of local variation in abundance at this density, highly decoupled from local abundance (i.e. because it serves as a territory marker) or that the gradient of landscape heterogeneity covered by the study sites falls within a value range of low variability in the index value due to a non-linear relationship with true abundance (e.g. positive quadratic).

In general, RAIs obtained by camera traps are sensitive to differences in detection probability that are related to choice of camera location and site characteristics, which need to be accounted for. In our study, this was reflected in the number of detection covariates retained after model selection (Table 2) as compared to other methods.

**Table 2:** Model results of the best model for each method in each session. Parameter estimates and associated standard errors (in brackets) are provided for each model. Asterisks indicate term significance in the model. For abbreviations see method section. The first half of the table contains environmental predictors at the plot level, the second half site specific control variables and other covariates of detection probability retained in the models. For brevity, we only report whether control variables were included in the model. Comprehensive parameter estimates are reported in Table B in S1 Appendix.

	Session A		Session B		Session C	
	**CT**	**Random Transect**	**CT**	**Square Scat Plot**	**CT**	**Linear features**
Intercept	2.16 * (0.24)	0.07 (0.12)	1.87 * (0.02)	1.34 * (0.08)	2.54 * (0.08)	0.36 (0.29)
**Shannon**	0.25 * (0.02)	0.31 * (0.12)	0.11 * (0.02)	**0.03** **(0.08)**	0.44 * (0.03)	**0.01** **(0.16)**
Elevation	-0.06 * (0.02)	-	-0.18 * (0.02)	-0.18 * (0.09)	-	-
Dist. Settle.	-	-	-0.05 * (0.02)	-	-	-
Prop. Settle	-	-	-	-	-0.22 * (0.03)	-
Prop. Forest	-	-	-	-	0.70 * (0.06)	-0.34 (0.19)
%Resistance	✓		✓			
Small trail	✓		✓			
Observer ID						✓
Ground cover					✓	
Type of feature					✓	
Round 1 vs. 2					✓	
Model R^2^	0.09	0.06	0.15	0.06	0.37	0.28

With regards to costs and effort, faeces count-based methods were cheaper than camera traps for an initial sampling session, but camera traps had much lower costs for long-term research or monitoring (i.e. 213–463% lower, depending on the method). When considering upscaling of wildlife monitoring to a larger number of studies sites, methods should either be less labour-intensive or, if labour-intensive, comparatively simple in order to make efficient use of volunteer field assistants [12]. Although the choice of larger scat plots removed the issue of excessive zeros in the data while retaining comparatively high precision (Table 1), this method was associated with significant field effort and cost (i.e. 3 man hours per plot). This may be prohibitive for application in large-scale monitoring or if monitoring of fox abundance is only one of several objectives of the study. Conversely, camera trap deployment was less time consuming (371–857% faster per plot than faeces-based methods; 233–516% when including post-processing time), strongly decreasing the number of personnel required and thus the dependency on volunteers. It is worth noting, however, that if cameras are set to record large numbers of pictures per session post-processing may become a significant cost. Also, camera traps are potentially subject to technical failure, manipulation and theft–especially in densely populated areas. Yet, camera traps remained the cheapest method in the long run even after accounting for theft.

In either case, the performance of faeces-count based methods in this study supports their use for studies aiming at paired testing of differences in local abundance between study sites of equal landscape composition and otherwise similar detection probability, rather than for studies with a design that requires accounting for influences of landscape composition on local abundance. In this context, camera trap-based methods appear to provide more flexible data as long as differences in detection probability due to camera setup and camera site can be standardized and accounted for. Such standardized camera data are also not subject to identification error or observer bias, while faeces-based methods are influenced by identification uncertainty (i.e. similarity to marten or domestic dog scat) and differences in search efficiency between observers. While faeces count-based methods are relatively easy to conduct with the help of trained volunteers (e.g. students, hunters), the effects of differences in observer qualification on scat detection rates and identification certainty are unknown and potentially difficult to quantify. Misidentification rates of expert observers were low in previous studies in our study area (e.g. 4.8% in [26]). In our study, we used multi-observer validation of the doubtful scats to account for differences in identification certainty, but inter-observer variation in detection rates remained impossible to quantify. Finally, camera traps can produce RAIs for a variety of species apart from the focal species, which may be useful for monitoring of multiple species, as long as assumptions of detection probability with regards to camera design (e.g. in our case detectability of the species on tracks) hold for all species within the study area [11]. While faeces-based methods are potentially capable of detecting other species as well, the relationship of scat frequency with true abundance as well as assumptions regarding scat placement and detectability need to be assessed on a case by case basis. Finally, another advantage of camera traps may be that the resulting RAI data (i.e. counts of detection events over time) could be used with one of the emerging methods to estimate absolute density without the need for individual recognition–such as the random encounter model [7], the Bayesian spatial count model for unmarked populations [8] or the recently proposed time- and space to event models [9]–but data analysis may be challenged by the need to fulfil method specific assumptions, some of which may be impractical to achieve. Accordingly, the choice of the appropriate method will depend on the environmental context and the objectives of the study under explicit consideration of the respective advantages and weaknesses of each method (Figure A in S1 Appendix).

## Supporting information

S1 Appendix Supporting information for Kämmerle et al. Methods for assessing small-scale variation in the abundance of a generalist mesopredator. (DOCX)

S1 Data Red fox RAIs and model data in three study sessions. (ZIP)
