# Supplementary material for: Methods for assessing small-scale variation in the abundance of a generalist mesopredator
Source: PLoS One. 2018 Nov 21;13(11):e0207545. doi: 10.1371/journal.pone.0207545 (PMC6248971; doi:10.1371/journal.pone.0207545)
Supplement: S1 Appendix — (DOCX) [file pone.0207545.s001.docx]

**Methods for assessing small-scale variation in the abundance of a generalist mesopredator**

Jim-Lino Kämmerle^1,2†^, Luca Corlatti^1^, Laura Harms^1^, Ilse Storch^1^

**Supplementary Material**

^1^Chair of Wildlife Ecology and Wildlife Management, University of Freiburg, Germany

^2^Forest Research Institute of Baden-Württemberg FVA, Freiburg, Germany

^†^Corresponding author: [lino.kaemmerle@wildlife.uni-freiburg.de](mailto:lino.kaemmerle@wildlife.uni-freiburg.de)

**Table A:** Characteristics of the methods in this study. Session A and B took place in montane forests and session C in agriculturally dominated lowlands. Variance estimates are provided for the raw data (naïve) and as obtained from the final models assuming a negative binomial distribution (negbin). Cost per plot is estimated as the sum of costs for material and personnel (excluding travel; see method section) and provided for both a first session and any consecutive session. Covariates used in the individual regression models are provided. Note that the methods have different units: Camera traps (CT): number of fox events over 21 days; Random and linear transects: number of scats per transect; Square scat plots: number of scats per plot.

|  | **Session A** | | | **Session B** | | | **Session C** | | | |  |  |
| --- | --- | --- | --- | --- | --- | --- | --- | --- | --- | --- | --- | --- |
|  | | | **CT** | **Random Transect** | | **CT** | **Square Scat Plot** | | **CT** | **Random Transect** | **Linear features** | |
| **Data collection** | | |  |  | |  |  | |  |  |  | |
| Time | | | 17.03. -12.05.2017 | 29.03. -10.05.2017 | | 15.03. -16.05.2018 | 03.04. -18.04.2018 | | Nov. 2017 Feb. 2018 | 20.11. -24.11.2017 | 06.02. -08.02.2018 | |
| Plot size | | | 250m radius | | | 500m x 500m | | | 1000m x 1000m | | | |
| Effort control | | | 1 camera / plot | 1300m / plot | | 1 camera / plot | 3 hours / plot | | 1 camera / plot | 1300m / plot | 1300m / plot | |
| Effort placement | | | Plot centre outwards | Random triangle on plot centre | | Plot centre outwards | Whole plot | | Plot centre outwards | Random triangle on plot centre | Along linear features | |
| Sample size | | | 132 | 114 | | 151 | 82 | | 38 + 39 | 40 | 40 | |
| **Data Properties*** | | |  |  | |  |  | |  |  |  | |
| Mean | | | 7.94 | 1.12 | | 4.38 | 3.88 | | 4.36 | 0.68 | 1.68 | |
| Median | | | 5.46 | 1.00 | | 2.17 | 3.00 | | 2.86 | 0.50 | 1.00 | |
| Var (naïve) | | | 81.4 | 2.10 | | 31.1 | 11.1 | | 45.7 | 0.64 | 4.02 | |
| Var (negbin) | | | 67.3 | 1.89 | | 32.5 | 8.21 | | 30.7 | - | 2.46 | |
| θ (negbin) | | | 1.20 | 1.65 | | 1.04 | 3.47 | | 1.08 | - | 3.56 | |
| α (negbin) | | | 0.83 | 0.61 | | 0.96 | 0.29 | | 0.93 | - | 0.28 | |
| CV | | | 1.03 | 1.23 | | 1.30 | 0.74 | | 1.27 | - | 0.93 | |
| Min | | | 0 | 0 | | 0 | 0 | | 0 | 0 | 0 | |
| Max | | | 59 | 7 | | 34 | 23 | | 50 | 3 | 9 | |
| % Zeros | | | 6.8 | 43.9 | | 17.8 | 4.9 | | 24.7 | 50.0 | 27.5 | |
| **Field effort** | | |  |  | |  |  | |  |  |  | |
| Hours / plot | | | 0.6 | 1.6 | | 0.6 | 3.1 | | 0.6 | 1.4 | 1.4 | |
| Nr. Observers | | | 1 | 6 | | 1 | 6 | | 1 | 3 | 3 | |
| Cost / plot (1^st^) | | | 270.4€ | 49.5€ | | 270.4€ | 94.5€ | | 270.4€ | 43.5€ | 43.5€ | |
| Cost / plot (2^nd^) | | | 20.4€ | 49.5€ | | 20.4€ | 94.5€ | | 20.4€ | 43.5€ | 43.5€ | |
| **Covariates used** | | |  |  | |  |  | |  |  |  | |
| Environmental | | | Shannon index  Elevation  Distance to Settlement | | | Shannon index  Elevation  Distance to Settlement | | | Shannon index  Proportion of settlement / plot  Proportion of forest / plot | | | |
| Detection | | | % resist.  Trail type | Observer | | % resist.  Trail type | Observer | | LF type  Ground C  Period | *No analysis* | Observer | |

**Variable abbreviations**: Shannon Index – the Shannon index of landcover types as an index for landscape heterogeneity; % resist. - % of ground around camera trap covered by structures that hinder fox movement; Observer – the ID of the observer collecting the data; Trail type – the type of trail (2 levels) at which the camera was installed; LF type – the type of linear landscape feature (4 levels) at which the camera was installed; Ground C – the type of ground cover at the camera site (5 levels); Period – the period of fieldwork, either November or February; *units of measurement are: CT – number of fox events per trap station and study period; Transects and scat plots – number of faeces found per transect unit.

**Table B:** Model results of the best model for each method in each session. Parameter estimates and associated standard errors (in brackets) are provided for each model. Asterisks indicate term significance in the model. For abbreviations see method section. The first half of the table contains environmental predictors at the plot level, the second half site specific control variables and other covariates of detection probability retained in the models.

|  | **Session A** | | **Session B** | | **Session C** | |
| --- | --- | --- | --- | --- | --- | --- |
|  | **CT** | **Random Transect** | **CT** | **Square Scat Plot** | **CT** | **Linear features** |
| Intercept | 2.16 *  (0.24) | 0.07  (0.12) | 1.87 *  (0.02) | 1.34 *  (0.08) | 2.54 *  (0.08) | 0.36  (0.29) |
| **Shannon** | **0.25 ***  **(0.02)** | **0.31 ***  **(0.12)** | **0.11 ***  **(0.02)** | **0.03**  **(0.08)** | **0.44 ***  **(0.03)** | **0.01**  **(0.16)** |
| Elevation | -0.06 *  (0.02) | - | -0.18 *  (0.02) | -0.18 *  (0.09) | - | - |
| Dist. Settle. | - | - | -0.05 *  (0.02) | - | - | - |
| Prop. Settle | - | - | - | - | -0.22 *  (0.03) | - |
| Prop. Forest | - | - | - | - | 0.70 *  (0.06) | -0.34  (0.19) |
| %Resistance | 0.22 *  (0.02) |  | 0.09 *  (0.02) |  |  |  |
| Small trail | -0.16 *  (0.04) |  | -0.91 *  (0.04) |  |  |  |
| Observer ID 2 |  |  |  |  |  | 0.54  (0.36) |
| Observer ID 3 |  |  |  |  |  | -0.72  (0.47) |
| Observer ID 4 |  |  |  |  |  | 0.51  (0.85) |
| Groundcover C2 |  |  |  |  | 0.48 * (0.08) |  |
| Groundcover C3 |  |  |  |  | 0.58 *  (0.08) |  |
| Groundcover C4 |  |  |  |  | -0.40*  (0.10) |  |
| Groundcover C5 |  |  |  |  | -0.70*  (0.12) |  |
| Feature type 2 |  |  |  |  | -2.28*  (0.13) |  |
| Feature type 3 |  |  |  |  | -1.26*  (0.09) |  |
| Feature type 4 |  |  |  |  | -0.51*  (0.07) |  |
| Round 2 vs. 1 |  |  |  |  | -0.60*  (0.06) |  |
| Model R² | 0.09 | 0.06 | 0.15 | 0.06 | 0.37 | 0.28 |

**Table C:** Number of pictures taken by camera traps in each session in relation to the number of fox pictures and fox events. The number of pictures reported is the number of pictures with larger mammals (i.e. all mammals ≥ squirrel) after deletion of empty images, small mammals and birds. Note that the data are not corrected for the duration of the sampling period (i.e. means fox events not equal to results in the manuscript).

|  | **Number images** | **Images / station** | **Number fox images** | **Fox images / station** | **Number fox events** | **Fox events / station** |
| --- | --- | --- | --- | --- | --- | --- |
| ***Session A*** | 18.802 | 142 | 4.313 | 33 | 1110 | 8.4 |
| ***Session B*** | 10.631 | 70 | 1.850 | 12 | 804 | 5.3 |
| ***Session C*** | 6.046 | 79 | 715 | 9 | 404 | 5.2 |


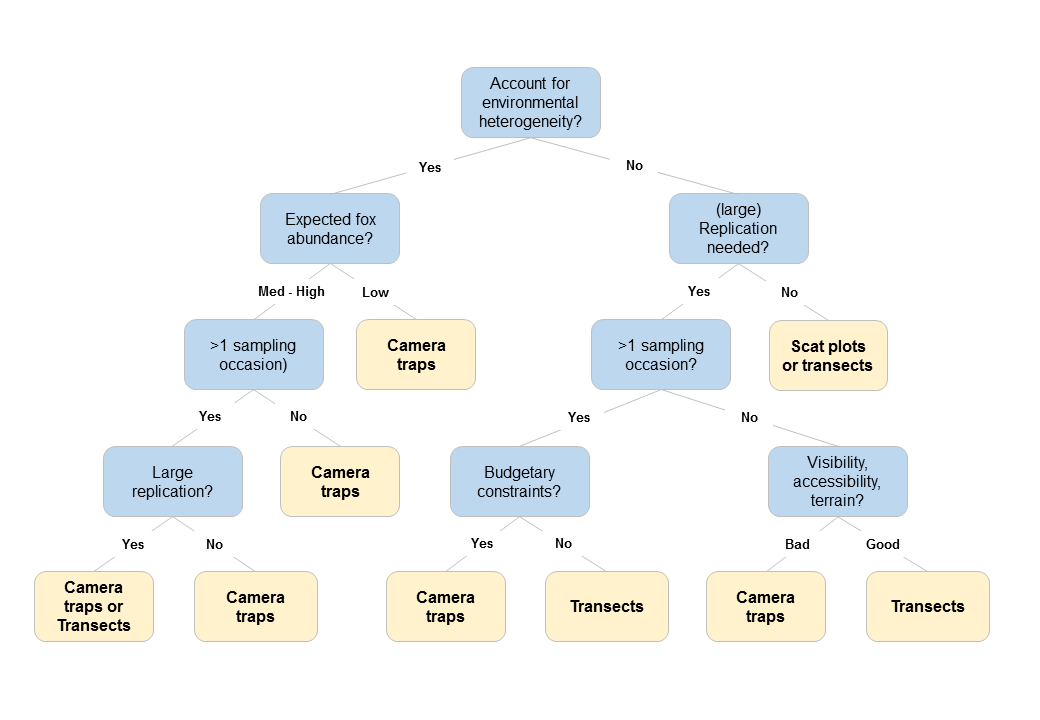


**Figure A**: Decision tree diagram for the appropriate choice of method based on the performance of the difference methods in our study.
